# Expanded safety experience with lenalidomide plus dexamethasone in relapsed or refractory multiple myeloma

**DOI:** 10.1111/j.1365-2141.2009.07728.x

**Published:** 2009-07

**Authors:** Christine Chen, Donna E Reece, David Siegel, Ruben Niesvizky, Ralph V Boccia, Edward A Stadtmauer, Rafat Abonour, Paul Richardson, Jeffrey Matous, Shaji Kumar, Nizar J Bahlis, Melissa Alsina, Robert Vescio, Steven E Coutre, Dennis Pietronigro, Robert D Knight, Jerome B Zeldis, Vincent Rajkumar

**Affiliations:** 1 Princess Margaret HospitalToronto, ON, Canada; 2 The Cancer Center at Hackensack University Medical CenterHackensack, NJ; 3 Weill Cornell Medical CollegeNew York, NY; 4 Center for Cancer and Blood DisordersBethesda, MD; 5 University of Pennsylvania Abramson Cancer CenterPhiladelphia, PA; 6 Indiana University Simon Cancer CenterIndianapolis, IN; 7 Dana-Farber Cancer Institute, Harvard Medical SchoolBoston, MA; 8 Rocky Mountain Cancer CentersDenver, CO; 9 Mayo ClinicRochester, MN, USA; 10 University of CalgaryCalgary, AB, Canada; 11 H. Lee Moffitt Cancer Center and Research InstituteTampa, FL; 12 University of California Los Angeles School of Medicine and Jonsson Comprehensive Cancer CenterLos Angeles, CA; 13 Stanford University School of MedicineStanford, CA; 14 Celgene CorporationSummit, NJ, USA

**Keywords:** multiple myeloma, relapsed/refractory, lenalidomide plus dexamethasone, combination therapy

## Abstract

Lenalidomide gained Food and Drug Administration (FDA) approval for treatment of patients with relapsed or refractory multiple myeloma (MM) in combination with dexamethasone in June 2006. In April 2005, the FDA and patient advocacy groups requested an expanded access programme to both provide lenalidomide to patients likely to benefit and obtain additional safety information. Relapsed/refractory MM patients received lenalidomide 25 mg/d (days 1–21) and dexamethasone 40 mg/d (days 1–4, 9–12, and 17–20 of cycles 1–4; days 1–4 only from cycle 5 onwards), in 4-week cycles until disease progression, study drug discontinuation, or lenalidomide approval. Of the 1438 patients enrolled, ∼60% were male, median age was 64 years, and 61·7% had Durie-Salmon stage III disease. Median time on study was 15·4 weeks (range: 0·1–49·1) and median dose was 25 mg. The most common adverse events (AEs) were haematological (49%), gastrointestinal (59%), and fatigue (55%). The most common grade ≥3 AEs were haematological (45%), fatigue (10%), and pneumonia (7%). The most common serious AEs were pneumonia (8%), pyrexia (4%), and deep-vein thrombosis (3%). Primary cause of death was disease progression (10%). Safety data confirmed known AEs of lenalidomide plus dexamethasone therapy in patients with relapsed/refractory MM.

The outcomes for many patients with multiple myeloma (MM) have been improved in the past few years with the introduction of the immunomodulatory drugs thalidomide and lenalidomide, and the proteasome inhibitor bortezomib, though the disease is usually still incurable (Kyle & Rajkumar, 2004; Anderson *et al*, 2007). Lenalidomide (Revlimid®; Celgene Corporation, Summit, NJ, USA) is a more potent analogue of thalidomide that is effective (with a good safety profile) in the treatment of patients with relapsed or refractory MM (Corral *et al*, 1999; Hideshima *et al*, 2000; Richardson *et al*, 2002, 2006; Dimopoulos *et al*, 2007; Weber *et al*, 2007), and transfusion-dependent International Prognostic Scoring System (IPSS)-defined Low- and Int-1-risk myelodysplastic syndromes (MDS) associated with a deletion 5q [del(5q)] chromosomal abnormality (List *et al*, 2006). On 29 June 2006, the US Food and Drug Administration (FDA) approved lenalidomide given in combination with dexamethasone for the treatment of patients with MM who had received prior therapy, based on the results from two parallel, randomized, double-blind, placebo-controlled phase III trials (Dimopoulos *et al*, 2007; Weber *et al*, 2007). In June 2007, the European Medicines Agency approved lenalidomide for the same indication. In pre-planned interim analyses of these two trials, patients treated with the lenalidomide and dexamethasone combination had a significantly longer time to disease progression compared with patients treated with dexamethasone alone. Based on these results, an Independent Data Safety Monitoring Board recommended unblinding of the studies so that all enrolled patients could have access to lenalidomide.

In April 2005, over a year before FDA approval and while lenalidomide therapy for relapsed or refractory MM was under review, the Oncology Division of the FDA, along with myeloma patient advocacy groups, requested that the manufacturer of lenalidomide, Celgene Corporation, establish an ‘expanded access programme’. This would make lenalidomide, in combination with dexamethasone, available to patients with relapsed or refractory MM with a high likelihood of benefit. On this basis, the safety study protocol MM-016 was initiated. Here we provide the safety analysis of patients with relapsed or refractory MM who participated in this expanded access trial.

## Patients and methods

### Patient eligibility and enrolment

The institutional review boards or ethics committees at each participating centre approved the study protocol and all patients provided written informed consent. The inclusion and exclusion criteria for the study were the same as for the pivotal phase III studies (MM-009 and MM-010) (Dimopoulos *et al*, 2007; Weber *et al*, 2007). Patients were eligible if they were aged ≥18 years, and had MM that had progressed after ≥2 cycles of anti-myeloma treatment or relapsed with progressive disease after treatment. Patients were required to have measurable levels of myeloma paraprotein in the serum (≥5·0 g/l) or urine (≥0·2 g secreted in a 24-h collection sample), and an Eastern Cooperative Oncology Group (ECOG) performance status score of ≤2. Prior thalidomide or radiation therapy was allowed. Radiation therapy initiated prior to or at baseline was permitted concurrent to study treatment, but all other anti-myeloma medication or other therapy had to be discontinued prior to the first study dose.

Women of childbearing potential had to have two negative pregnancy tests before starting the study treatment, and had to agree to use two reliable methods of contraception simultaneously or to practise complete abstinence from heterosexual intercourse from 28 d before the start of lenalidomide treatment until 28 d after treatment discontinuation. While participating in the study, and for at least 28 d after treatment discontinuation, men (including those who had undergone a successful vasectomy) had to agree to use a latex condom during sexual contact with women of childbearing potential. Participating men and women had to agree not to donate blood (and men also not to donate semen or sperm) during the study, and for at least 28 d after treatment discontinuation.

Patients were excluded from the study if they had: a prior history of malignancy other than MM, except for basal cell or squamous cell skin carcinoma or carcinoma *in situ* of the cervix or breast (unless the patient had been free of disease for at least 1 year); known hypersensitivity to thalidomide or dexamethasone; prior history of uncontrollable side-effects to dexamethasone treatment; development of desquamating rash while taking thalidomide; or neuropathy grade ≥2. Patients with any of the following laboratory abnormalities were also excluded from the study: those with an absolute neutrophil count of <1·0 × 10^9^/l; those with a platelet count of <75 × 10^9^/l (for patients in whom <50% of bone marrow nucleated cells were plasma cells), or a platelet count of <30 × 10^9^/l (for patients in whom ≥50% of bone marrow nucleated cells were plasma cells); serum creatinine >221 μmol/l; serum aspartate aminotransferase (AST) or alanine aminotransferase (ALT) levels >3× upper limit of normal; and serum total bilirubin >34·2 μmol/l.

Patients were enrolled in the study starting September 2005. Accrual in the USA was terminated following FDA approval of the MM indication in June 2006. Enrolment is continuing in Canada and is planned to continue until the drug is approved by the Canadian regulatory authorities. This report is a summary of the trial results from initiation on 6 September 2005 to termination of the US involvement on 21 September 2006.

### Treatment schedule and assessments

Patients were instructed to self-administer oral lenalidomide at a starting dose of 25 mg/d for 21 d, and dexamethasone 40 mg/d on days 1–4, 9–12, and 17–20. Each cycle was repeated every 28 d. Beginning with cycle 5, the dexamethasone schedule was reduced to 40 mg/d for days 1–4, every 28 d. Treatment was continued as tolerated, until disease progression or unacceptable adverse events as per the dose modification guidelines. After baseline evaluation at study entry, patients were seen at study visits every 2 weeks for the first three treatment cycles, and then every 4 weeks until documentation of disease progression or if treatment was discontinued for any reason.

Although the investigators assessed the patients for disease progression, there was no formal efficacy evaluation performed in this study. Safety evaluations included: vital signs; haematology; serum chemistries; serum thyroid-stimulating hormone; thyroid function (T_3_, T_4_); electrocardiogram; serum and urine beta-human chorionic gonadotrophin (for female patients of childbearing potential only); and adverse events. The severity of adverse events was graded according to the National Cancer Institute Common Terminology Criteria for Adverse Events (National Cancer Institute, 2006). An internal data monitoring committee reviewed ongoing safety data throughout the study. The members of the committee included representatives from regulatory affairs and drug safety, a lead product safety physician, a biostatistician, and three medical officers (one an oncologist).

Lenalidomide was supplied by the manufacturer in bottles containing 21 (25-mg or 5-mg) capsules. Commercial supplies of dexamethasone were used. Antithrombotic therapy, such as daily aspirin, was recommended as prophylaxis for deep-vein thrombosis (DVT); daily prophylaxis with antibiotics was recommended for all patients; and erythropoietin was recommended for chronic transfusion-dependent anaemia, but none of these was mandated. Other therapies considered necessary for the well-being of the patient (e.g. analgesics, antihistamines, growth factors, and transfusions of red blood cells, platelets, or fresh-frozen plasma) were allowed at the discretion of the investigator.

### Dose modifications

Lenalidomide doses were interrupted for: grade 3 or 4 neutropenia; thrombocytopenia with a platelet count of <30 × 10^9^/l; grade 2 allergic reaction or hypersensitivity; hyperthyroidism or hypothyroidism of grade ≥2; grade 2 neuropathy; and other lenalidomide-related grade 3 or 4 adverse events. Treatment was resumed at a lower dose level after the adverse event improved to grade 2 or better. Lenalidomide treatment was discontinued for grade ≥3 rash/desquamation, grade 3 or 4 allergic reaction or hypersensitivity, and grade 3 or 4 neuropathy. For patients with grade ≥3 venous thrombosis or embolism, the dose was interrupted and anticoagulation therapy was started. Lenalidomide treatment was to be restarted at the same dose level at the physician’s discretion.

The protocol included four lenalidomide dose reduction levels. Level −1 included maintenance of the 25-mg starting dose, supplemented by granulocyte colony-stimulating factor according to the American Society of Clinical Oncology (ASCO) guidelines (Ozer *et al*, 2000). This level was recommended for patients with grade 3 or 4 neutropenia only. For all other adverse events requiring lenalidomide dose reduction, the dose reductions were Level −2 (15 mg), Level −3 (10 mg), and Level −4 (5 mg). Lenalidomide treatment was discontinued for patients who could not tolerate dose Level −4.

Dexamethasone doses were interrupted or reduced in response to dyspepsia, gastric or duodenal ulcers, gastritis, oedema, confusion or mood alterations, muscle weakness, hyperglycaemia, and acute pancreatitis. There were three dexamethasone dose reduction levels: 40 mg/d for 4 d every 2 weeks; 40 mg/d for 4 d every 4 weeks; and 20 mg/d for 4 d every 4 weeks.

For both lenalidomide and dexamethasone, dose escalation was not permitted after dose reduction.

### Statistical design and analysis

The primary objective of this trial was to provide lenalidomide to patients with a high likelihood of benefit. The secondary objective was to obtain additional safety data. The study end-point was safety, including assessment of type, frequency, severity, and relationship of side-effects to study treatment. Data from all patients who received at least one dose of lenalidomide were included in the safety analyses. Continuous demographic and baseline variables were summarized using descriptive statistics, whereas categorical variables were summarized in frequency tabulations. The median exposure to lenalidomide was calculated as the average dose over all the days between the first and last dose date, including the 7 d off-drug in each 28-d cycle and any interruption periods. Thus a dose exposure of 18·75 mg/d was 100% of the scheduled daily dose of 25 mg for 21 d of every 28-d cycle with no interruption.

## Results

Between 6 September 2005 and 21 September 2006, a total of 1438 patients from 69 centres in the USA and Canada were enrolled. Patient characteristics are summarized in Table I. The median age was 64 years (range: 29–91 years). Prior treatments included thalidomide, alkylators, bortezomib, high-dose chemotherapy/stem cell transplantation, and anthracycline. The median time on study was 15·4 weeks (range: 0·1–49·1 weeks). The median exposure to lenalidomide was 18·8 mg/d (range: 1·7–28·6 mg), over a median of 15·1 weeks (range: 0·1–49·1 weeks). This was 100% of the scheduled daily dose of lenalidomide 25 mg for 21 d. A total of 424 (29·5%) patients had at least one dose reduction (dose level −2 through −4), with 306 patients requiring one dose reduction, 89 patients requiring two dose reductions, 28 patients requiring three dose reductions, and one patient requiring four dose reductions (the first dose reduction level for this patient was 20 mg). The adverse events most commonly causing dose reduction were thrombocytopenia (1·6%), neutropenia (1·5%), and pneumonia (1·3%).

**Table I:** Patient demographics and disease characteristics (*N*=1438).

	Patients	
Characteristic	*n*	%
Age, years		
Median	64	
Range	29–91	
Sex		
Male	863	60
Female	575	40
Race*		
White	1191	82·8
Black	154	10·7
Hispanic	47	3·3
Asian/Pacific Islander	26	1·8
American Indian/Alaska native	2	0·1
Other	18	1·3
Stage of disease (Durie-Salmon)		
I	176	12·2
II	358	24·9
III	887	61·7
Missing	17	1·2
Prior multiple myeloma therapy†		
Alkylators	846	58·8
Anthracycline	566	39·4
High-dose chemotherapy/stem cell transplantation	758	52·7
Bortezomib	824	57·3
Vinca alkaloid	548	38·1
Thalidomide	1094	76·1
Other	865	60·2
Medical history†		
Neuropathy	958	66·6
Venous thromboembolism	227	15·8

* Percentages may add up to more than 100% as patients were allowed to select more than one race.

† Percentages may add up to more than 100% as more than one response was allowed.

### Adverse events

The most common adverse events (all grades) were haematological, gastrointestinal, fatigue, and muscle cramps (Table II). Haematological events (neutropenia, thrombocytopenia, leucopenia, and anaemia) were manageable with dose reductions and resulted in only 3·75% of patients discontinuing lenalidomide treatment. Hypokalaemia, hyperglycaemia not otherwise specified (NOS), and blurred vision were noted in less than 10% of patients. The most common grade ≥3 adverse events were haematological, fatigue, and pneumonia (Table III). The most common grade 4 adverse events were thrombocytopenia (7·1%), neutropenia (5·2%), anaemia (1·2%), and pneumonia (1·0%). At least one grade 3 or 4 adverse event was reported in 69·7% of patients. The most common serious adverse events were pneumonia, pyrexia, DVT, and thrombocytopenia (Table IV).

**Table IV:** Most common serious adverse events (*N*=1438).

	Patients	
Serious adverse event	*n*	%
Pneumonia	117	8·1
Pyrexia	54	3·8
Deep-vein thrombosis	43	3·0
Thrombocytopenia	42	2·9
Dehydration	36	2·5
Febrile neutropenia	36	2·5
Anaemia	35	2·4
Dyspnoea	29	2·0

**Table III:** Most common grade 3 or 4 adverse events due to any cause reported in >2·0% of patients (*N*=1438).

	Patients	
Adverse event	*n*	%
Patients with ≥1 grade 3 or 4 adverse event	1002	69·7
Haematological		
Neutropenia	315	21·9
Thrombocytopenia	195	13·6
Anaemia NOS	111	7·7
Febrile neutropenia	35	2·4
General		
Fatigue	149	10·4
Asthenia	53	3·7
Infections		
Pneumonia NOS	102	7·1
Metabolic		
Hyperglycaemia NOS	61	4·2
Dehydration	38	2·6
Musculoskeletal		
Back pain	39	2·7
Muscle weakness NOS	37	2·6
Investigations		
Neutrophil count decreased	31	2·2
Respiratory		
Dyspnoea NOS	57	4·0
Venous thromboembolism		
Deep-vein thrombosis	65	4·5

NOS, not otherwise specified.

**Table II:** Most common grade 1–4 adverse events due to any cause reported in ≥10·0% of patients (*N*=1438).

	Patients	
Adverse event	*n*	%
Patients with ≥1 adverse event	1404	97·6
General		
Fatigue	796	55·4
Asthenia	220	15·3
Pyrexia	215	15·0
Peripheral oedema	202	14·0
Gastrointestinal		
Constipation	341	23·7
Diarrhoea NOS	297	20·7
Nausea	272	18·9
Musculoskeletal		
Muscle cramp	338	23·5
Back pain	190	13·2
Arthralgia	145	10·1
Nervous system		
Dizziness	170	11·8
Neuropathy NOS	145	10·1
Haematological		
Neutropenia	425	29·6
Anaemia NOS	340	23·6
Thrombocytopenia	306	21·3
Respiratory		
Dyspnoea NOS	235	16·3
Cough	223	15·5
Infections		
Pneumonia NOS	155	10·8
Upper respiratory tract infection NOS	144	10·0
Psychiatric		
Insomnia	286	19·9
Skin		
Rash NOS	186	12·9
Metabolic		
Anorexia	147	10·2

NOS, not otherwise specified.

Neuropathic symptoms (neuropathy NOS, peripheral neuropathy NOS, peripheral sensory neuropathy, peripheral motor neuropathy, and polyneuropathy NOS) were reported by 18·2% of patients on the study at some point during their treatment. The majority (224 of 263 [85·2%] patients) had grade 1 or 2 events. Of the 18·2% patients who experienced neuropathic symptoms, 73·9% had a prior history. Two thirds (67%) of all patients on study had a prior history of neuropathy.

Venous thromboembolic events (VTEs) of all grades occurred in 8·3% of patients with a median duration of 12 d (range: 1–113 d). VTEs included DVT (6·1%), pulmonary embolism (1·5%), thrombosis (0·8%), jugular vein thrombosis (0·1%); thrombosis (0·9%; thrombosis, phlebothrombosis), unspecified phlebitis (0·3%), and unspecified embolism (0·2%). Grades 3/4/5 VTEs occurred in 5·8% of patients. Arterial thromboembolic events (ATEs) occurred in 2·9% of patients. ATEs included myocardial infarction (0·5%), transient ischaemic attack (0·4%), cerebrovascular accident (0·4%), and thrombotic stroke (0·1%). Only 1·8% of these patients had an grade ≥3, although 6 (0·4%) patients experienced grade 5 ATEs.

### Incidence of dose reductions and discontinuations due to adverse events

Overall, 18·5% of patients enrolled in the trial discontinued treatment with lenalidomide due to adverse events. The most common causes of lenalidomide discontinuation due to adverse events were thrombocytopenia, neutropenia, pneumonia, and sepsis (Table V). Other reasons for treatment discontinuation included commercial availability of the drug in the USA (46·7%), disease progression (18·5%), death (5·1%), and withdrawal of consent (4·4%). Neutropenia (17·8%), thrombocytopenia (9·2%), fatigue (8·3%), pneumonia (3·5%), pyrexia (3·5%), and anaemia (3·3%) were the most common adverse events that led to dose reductions, interruptions, or discontinuation. The median time to the first dose reduction, interruption, or discontinuation was 36 d (range: 1–282 d).

**Table V:** Most common adverse events leading to treatment discontinuation due to any cause reported in >0·5% of patients (*N*=1438).

	Patients	
Adverse event	*n*	%
Patients with ≥1 adverse event leading to discontinuation of study drug	266	18·5
Thrombocytopenia	23	1·6
Neutropenia	21	1·5
Pneumonia NOS	19	1·3
Sepsis NOS	14	1·0
Fatigue	11	0·8
Pancytopenia	11	0·8
Anaemia NOS	10	0·7
Asthenia	10	0·7
Multiple myeloma	9	0·6
Rash NOS	9	0·6
Acute renal failure	9	0·6
Dyspnoea NOS	8	0·6
Pyrexia	8	0·6

NOS, not otherwise specified.

### Deaths

Overall, 216 deaths (15%) were reported in the trial, with 125 deaths (8·7%) occurring within 30 d from the last dose. The most frequent causes of death were disease progression (10·2%) and adverse events (4·3%); 1% of deaths were due to adverse events related to the study drug.

## Discussion

Thalidomide emerged as an anticancer agent after investigators demonstrated its antineoplastic activity in haematological malignancies and, in particular, for the treatment of patients with MM (Singhal *et al*, 1999; Rajkumar *et al*, 2000, 2002, 2006; Barlogie *et al*, 2001; Weber *et al*, 2003). Lenalidomide was developed to further enhance efficacy while avoiding the teratogenicity, sedation, peripheral neuropathy, and severe constipation associated with thalidomide therapy (Richardson *et al*, 2002, 2006; Rajkumar *et al*, 2005; Dimopoulos *et al*, 2007; Weber *et al*, 2007).

The expanded access trial permitted lenalidomide plus dexamethasone to be available to patients in the USA and Canada while the drug awaited FDA approval for use in patients with MM. During the course of this trial, the FDA approved the use of the drug for transfusion-dependent IPSS Low- or Int-1-risk MDS associated with a del(5q) chromosomal abnormality. This trial was continued as the daily dose of lenalidomide used for the treatment of MDS was 10 mg, and that for MM is 25 mg. This trial provides a model of how regulatory agencies, advocacy groups, healthcare providers, and industry can work together to quickly provide an effective treatment to a large number of patients in need, while a regimen with clearly demonstrated safety and activity awaits approval.

The type and frequency of adverse events from over 1400 patients with MM enrolled in this trial are consistent with results from the phase III studies used to register the drug in the USA and Europe. Compared with the MM-009 study, a similar number of patients discontinued treatment due to adverse events (19·8% vs. 18·5%, respectively) (Weber *et al*, 2007).

The most commonly observed grade 3 or 4 adverse events in this study occurred at a similar frequency as those reported for the pivotal MM-009 and MM-010 studies (Dimopoulos *et al*, 2007; Weber *et al*, 2007). Among the MM-009 and MM-010 patients who were treated with lenalidomide and dexamethasone, thrombosis occurred in 23% of patients who received concomitant erythropoietic therapy, such as epoetin alfa or darbepoetin, compared to 5% who did not (Knight *et al*, 2006). The lower frequency of thromboembolic events that occurred in this trial may be attributed to the strong recommendation that daily aspirin or other thromboprophylactic therapy continued to be used, which has been shown to decrease the incidence of VTE without bleeding complications (Baz *et al*, 2005; Rajkumar *et al*, 2005, 2006; Zonder *et al*, 2006).

The majority of patients (67%) on this trial had a prior history of neuropathy. Most neuropathy observed in this trial was grade 1 or 2. Although 18·2% of patients reported the occurrence of neuropathy, it is unclear if this represented residual neurological damage from prior therapies associated with neurological damage, such as thalidomide, vincristine, and bortezomib (Richardson *et al*, 2003, 2005; Glasmacher *et al*, 2006).

As with other studies of lenalidomide, most adverse events that occurred were well managed by lenalidomide dose reductions or interruptions, and symptom-control medications (according to study protocol).

In conclusion, the safety profile of lenalidomide therapy in conjunction with dexamethasone for relapsed or refractory MM in an expanded access programme is consistent with the two randomized, placebo-controlled trials that were the basis for US and European approval.
